# Differential therapeutic effects of atomoxetine and methylphenidate in childhood attention deficit/hyperactivity disorder as measured by near-infrared spectroscopy

**DOI:** 10.1186/s13034-017-0163-6

**Published:** 2017-05-12

**Authors:** Yoko Nakanishi, Toyosaku Ota, Junzo Iida, Kazuhiko Yamamuro, Naoko Kishimoto, Kosuke Okazaki, Toshifumi Kishimoto

**Affiliations:** 1 Department of Psychiatry, Nara Medical University School of Medicine, 840 Shijo-cho Kashihara, Nara, 634-8522 Japan; 2 Faculty of Nursing, Nara Medical University School of Medicine, 840 Shijo-cho Kashihara, Nara, 634-8522 Japan

**Keywords:** Attention-deficit/hyperactivity disorder, Near-infrared spectroscopy, Functional neuroimaging, Atomoxetine, Methylphenidate

## Abstract

**Background:**

The stimulant methylphenidate (MPH) and the nonstimulant atomoxetine (ATX) are the most commonly-prescribed pharmacological treatments for attention deficit/hyperactivity disorder (ADHD). However, the drug-specific mechanism of action on brain function in ADHD patients is not well known. This study examined differences in prefrontal hemodynamic activity between MPH and ATX in children with ADHD as measured by near-infrared spectroscopy (NIRS) using the Stroop color-word task.

**Methods:**

Thirty children with ADHD participated in the present study. We used 24-channel NIRS (ETG-4000) to measure the relative concentrations of oxyhemoglobin in the frontal lobes of participants in the drug-naïve condition and those who had received MPH (n = 16) or ATX (n = 14) for 12 weeks. Measurements were conducted every 0.1 s during the Stroop color-word task. We used the ADHD RS-IV-J (Home Version) to evaluate ADHD symptoms.

**Results:**

Treatment with either MPH or ATX significantly reduced ADHD symptoms, as measured by the ADHD RS-IV-J, and improved performance on the Stroop color-word task in terms of number of correct words. We found significantly higher levels of oxyhemoglobin changes in the prefrontal cortex of participants in the ATX condition compared with the values seen at baseline (pre-ATX). In contrast, we found no oxyhemoglobin changes between pre- and post-treatment with MPH.

**Conclusions:**

The present study suggests that MPH and ATX have differential effects on prefrontal hemodynamic activity in children with ADHD.

## Background

Attention-deficit/hyperactivity disorder (ADHD) is one of the most commonly diagnosed neurodevelopmental disorders in children with lifelong deficits in a wide range of executive functions [1]. ADHD symptoms are thought to arise from dysregulation of prefrontal and subcortical catecholamine neurotransmission [2, 3]. The stimulant methylphenidate (MPH) and the nonstimulant atomoxetine (ATX) are the most frequently prescribed drugs for the treatment of ADHD. Both drugs are known to reduce clinical ADHD symptoms. The common mechanism of both drugs is that they modulate dopamine (DA) and norepinephrine (NE) neurotransmission [4]. Small changes in DA or NE concentration affect networks of pyramidal cells in the prefrontal cortex (PFC), which regulates and sustains attention [5]. It is believed that the therapeutic effects of both medications occur primarily in the PFC [5], although the exact mechanisms of their actions are unclear.

Methylphenidate acts as an indirect DA agonist, inhibiting DA reuptake by occupying the DA transporter [6]. MPH has also shown to block the norepinephrine (NE) transporter in NE transporter-rich regions, including the PFC [7]. In rodent studies, MPH has been shown to enhance the extracellular levels of both DA and NE [8]. In contrast, although ATX is a selective NE reuptake inhibitor, it also inhibits DA reuptake in the PFC. Therefore, while it does not increase DA in the striatum, it increases both DA and NE in the prefrontal cortex [8]. The partially overlapping pharmacologic profiles of these medications suggest both similarities and differences in their therapeutic mechanisms of action. In the meta-analysis focused on the comparison between MPH and ATX, MPH showed a higher response rate compared to ATX [9]. In a randomized study directly comparing MPH and ATX in adults with ADHD, the effects on executive functions were generally similar, although there was a suggestion that ATX might show a slight benefit to the immediate-release MPH in terms of improving spatial planning [10]. However, another head-to-head study comparing the two drugs found that only osmotically-released MPH improved set-shifting and verbal fluency, although osmotically-released MPH and ATX both improved executive function generally in children and adolescents with ADHD [11]. Distinct underlying pharmacological mechanisms may cause these practical differences. There are few neuroimaging studies that examined these differences [12, 13]. Cubillo et al. showed that ATX upregulated and normalized right dorsolateral prefrontal cortex under activation measured by functional magnetic resonance imaging (fMRI), while MPH upregulated left inferior frontal cortex activation [13].

Near-infrared spectroscopy (NIRS) enables the noninvasive detection of neural activity near the surface of the brain using near-infrared light [14, 15]. It measures alterations in oxygenated hemoglobin ([oxy-Hb]) and deoxygenated.

Hemoglobin ([deoxy-Hb]) concentrations in micro-blood vessels on the brain surface. Local increases in [oxy-Hb] and decreases in [deoxy-Hb] are indicators of cortical activity [15, 16]. In animal studies, [oxy-Hb] is the most sensitive indicator of regional cerebral blood flow because the direction of change in [deoxy-Hb] is determined by the degree of changes in venous blood oxygenation and volume [17]. Therefore, we decided to focus on changes in [oxy-Hb]. Furthermore, changes in [oxy-Hb] have been associated with changes in regional cerebral blood volume, using a combination of NIRS and positron emission tomography (PET) measurements [18, 19]. NIRS is a neuroimaging modality that is especially suitable for psychiatric patients for the following reasons [20]. First, because NIRS is relatively insensitive to motion artifact, it can be used in experimental scenarios in which motion may occur, such as while assessing participants who are prone to vocalization. Second, participants can be examined in a natural sitting position, without any surrounding distractions such as fMRI. Third, the cost is much lower than that of other neuroimaging modalities and the setup is very easy. Fourth, the high temporal resolution of NIRS is useful in characterizing the time course of prefrontal activity in people with psychiatric disorders [21, 22]. Fifth, functional studies of pediatric patients using single-photon emission computed tomography (SPECT) and PET are rare due to restrictions regarding the use of radioactive materials in young individuals. Accordingly, NIRS has been used to assess brain function in people with many types of psychiatric disorders, including schizophrenia, bipolar disorder, post traumatic disorder, obsessive–compulsive disorder, and ADHD [20–28].

In pediatric ADHD, reduced prefrontal hemodynamic response has been measured by NIRS [23, 29, 30]. Negoro et al. examined prefrontal hemodynamic response during the Stroop color-word task in 20 children with ADHD and 20 healthy age- and sex-matched controls. They found that the oxy-Hb changes in the inferior prefrontal cortex in the control group were significantly larger than those in the ADHD group during the Stroop color-word task [23]. In an NIRS study of medication, Ota et al. examined the effects of a clinical dose of ATX on changes in prefrontal hemodynamic response during the Stroop color-word task in pediatric ADHD. They found that ATX induced an intensified prefrontal hemodynamic response [31]. In another NIRS study, Araki et al. found that the oxy-Hb concentration in the right dorsolateral PFC in the post-ATX condition was significantly increased compared to the pre-ATX condition during a continuous performance task [32]. Despite several NIRS studies with ADHD, only a few studies have examined the therapeutic effects of medication. Moreover, no studies have compared MPH with ATX directly. In this study, we examined the drug-specific effects of a clinical dose of either MPH or ATX on frontal activation as measured by NIRS in a cohort of medication-naïve pediatric ADHD subjects. We used the Stroop color-word task to assess inhibitory control and selective attention. As outlined above, there are distinct underlying pharmacological mechanisms associated with MPH and ATX. Therefore, we hypothesized that there might be a differential hemodynamic response across MPH and ATX.

## Methods

### Participants

Thirty patients aged 6–14 years and diagnosed with ADHD according to the DSM-5 criteria [33] participated in the present study. Participants had no history of treatment for a developmental disorder, and had consulted an experienced pediatric psychiatrist at the Department of Psychiatry at Nara Medical University. These participants underwent a standard clinical assessment comprising a psychiatric evaluation, a semi-structured diagnostic interview (the kiddie schedule for affective disorders and schizophrenia for school-age children-present and lifetime version [34]), and a medical history assessment. Two experienced pediatric psychiatrists confirmed the diagnosis of ADHD according to the DSM-5 criteria [33]. Intellectual level was assessed using the Wechsler intelligence scale for children-fourth edition (WISC-IV), and individuals with full-scale IQ (FIQ) scores below 70 were excluded. We also excluded those who presented with a comorbid Axis I diagnosis, a neurological disorder, a head injury, a serious medical condition, or a history of substance abuse/dependence because these influenced the prefrontal hemodynamic response [20–22, 24, 26, 35, 36]. In total, 30 participants with ADHD who had no previous medication history were enrolled in the present study. All participants were right-handed and of Japanese descent.

We used NIRS to measure the relative concentrations of oxy-Hb in participants in the drug-naïve condition (pre-treatment) and after 12 weeks of treatment with either osmotically released MPH (n = 16) or ATX (n = 14) (post-treatment). The participants were assigned either MPH or ATX by the decision of an experienced pediatric psychiatrist. All measurements were conducted at the same time of day (10.00–11.00 h). All the participants were MPH and ATX naïve and started to take MPH 18 mg/day or ATX 0.5 mg/kg/day in the morning, respectively. They were titrated up as needed to the lowest effective dose by the decision of an experienced pediatric psychiatrist every 2 weeks. The mean dose of MPH was 0.87 mg/kg (SD = 0.23), and the mean dose of ATX was 1.30 mg/kg (SD = 0.44). The characteristics of the participants are shown in Table 1. This study was approved by the Institutional Review Board at Nara Medical University. Written informed consent was obtained from all participants and/or their parents prior to the study.

**Table 1:** Participant characteristics

	MPH (n = 16)		ATX (n = 14)		p value
	Mean	SD	Mean	SD	
Sex (male/female)^a^	14/2		11/3		0.642
Age (years)	8.19	2.46	9.50	2.03	0.125
Medication dose (mg/kg)	0.87	0.23	1.30	0.44	NA
FIQ (WISC-IV)	94.19	13.46	96.64	14.43	0.634
ARF	30.63	10.65	32.29	13.46	0.709
ARI	16.75	5.52	18.29	6.84	0.502
ARH	13.88	6.72	14.00	7.99	0.963
SCWC-1	18.31	7.66	25.86	8.76	0.018
SCWC-2	19.81	9.56	29.36	11.11	0.017
SCWC-3	21.19	9.17	25.36	10.35	0.252

*MPH* methylphenidate, *ATX* atomoxetine, *NA* not applicable, *FIQ* full-scale IQ, *WISC*-*IV* Wechsler Intelligence Scale for children-fourth edition, *ARF* ADHD RS IV-J full scores, *ARI* ADHD RS IV-J inattention subscale scores, *ARH* ADHD RS IV-J hyperactivity subscale scores, *SCWC*-*1* Stroop color-word task number of correct answers first time, *SCWC*-*2* Stroop color-word task number of correct answers second time, *SCWC*-*3* Stroop color-word task number of correct answers third time

^a^The Chi square test was used; otherwise t-tests were used

### Assessment of ADHD symptoms

We used the ADHD Rating Scale-IV-Japanese version (ADHD RS-IV-J) (Home Version) [37] to evaluate ADHD symptoms in the participants. A higher ADHD RS-IV-J score is associated with more severe ADHD symptoms. All participants underwent ADHD RS-IV-J assessment pre- and post-treatment which were rated by parents (Table 3).

### The Stroop color-word task

The traditional Stroop task was combined with the word-reading task, incongruent color-naming task, and the color-naming task. However, we reconstructed the Stroop task according to previously described methods [38]. The Stroop color-word task consisted of two pages stapled together: each page had 100 items in five columns of 20 items each and the page size was 210 × 297 mm. On the first page, the words RED, GREEN, and BLUE were printed in black ink. On the second page, the words RED, GREEN, and BLUE were printed in red, green, or blue ink, with the limitation that the word meaning and ink color could not match. The items on both pages were randomly distributed, with the exception that no item could appear directly after the same item within a column. Before the task, the examiners instructed the participants as follows: ‘This is to test how quickly you can read the words on the first page, and say the colors of the words on the second page. After we say “begin”, please read the words in the columns, starting at the top left, and say the words/colors as quickly as you can. After you finish reading the words in the first column, go on to the next column, and so on. After you have read the words on the first page for 45 s, we will turn the page. Please repeat this procedure for the second page.’ The entire Stroop color-word task sequence consisted of three cycles of 45 s spent reading the first page and 45 s spent reading the second page (the color-word task). The task ended with 45 s spent reading the first page, which we designated as the baseline task. We recorded the number of correct answers in each cycle, and refer to them as follows:

Stroop color-word task number of correct answers first time (SCWC-1), second time (SCWC-2), and third time (SCWC-3). Examiners who were blind to the diagnoses of the participants administered the Stroop color-word task. The Stroop task used in this study was different from the traditional Stroop task. We made the Stroop color-word task simple because the participants were school-aged children. Furthermore, we excluded the color-naming task (part of the traditional Stroop task) because we wanted to have only two tasks (baseline task and activation task) for our NIRS study.

### NIRS measurements

**Fig. 1:**
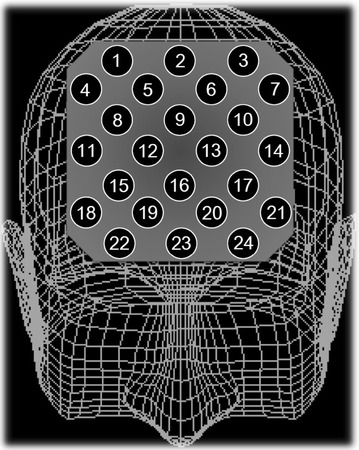
Location of the 24 channels on the near-infrared spectroscopy instrument

We measured [oxy-Hb] using a 24-channel NIRS machine (Hitachi ETG-4000, Hitachi Medical Corporation, Tokyo, Japan). We measured the absorption of two wavelengths of near-infrared light (760 and 840 nm). [Oxy-Hb] was calculated as previously described [39]. The inter-probe intervals of the machine were 3.0 cm, and previous reports have established that the machine measures at a point 2–3 cm beneath the scalp, that is, the surface of the cerebral cortex [36, 40]. The participants were asked to adopt a natural sitting position for the NIRS measurement. The distance between the participants’ eyes and the paper on which items were listed was between 30 and 40 cm. The NIRS probes were placed on the scalp over the prefrontal brain regions, and arranged to measure the relative changes in Hb concentration at 24 measurement points that made up an 8 × 8-cm^2^. The lowest probes were positioned along the Fp1–Fp2 line according to the international 10/20 system commonly used in electroencephalography. The correspondence between the probe positions and the measurement points in the cerebral cortex were confirmed by superimposing the probe positions onto a three-dimensionally reconstructed cerebral cortex of a representative participant in the control group, obtained via MRI (Fig. 1). The absorption of near-infrared light was measured with a time resolution of 0.1 s. The data were analyzed using the ‘integral mode’: the pre-task baseline was determined as the mean across the 10 s just before the task period, the post-task baseline was determined as the mean across the 25 s immediately after the task period, and linear fitting was performed on the data between the two baselines. Moving average methods were used to exclude short-term motion artifacts in the analyzed data (moving average window, 5 s). We attempted to exclude motion artifacts by closely monitoring artifact-evoking body movements, such as neck movements, biting, and blinking (identified as being the most influential in a preliminary artifact-evoking study), and by instructing the participants to avoid these movements during the NIRS measurements. Examiners were blind to the treatment condition of the participants.

### Statistical analysis

We used the Chi square (χ^2^) test to examine group differences for categorical variables (e.g. gender). Clinical variables with a normal distribution were compared using Student’s t tests. Correlations between SCWC and characteristics of the subjects were tested with Spearman’s correlation test. For statistical comparison of the participant characteristics between the pre- and post-treatment conditions, we used a two-tailed paired *t* test. Specifically, we compared oxy-Hb changes between the pre- and post-treatment conditions. To conduct a more detailed comparison of oxy-Hb changes along the time course of the task, we used MATLAB 6.5.2 (Mathworks, Natick, MA, USA) and Topo Signal Processing type-G version 2.05 (Hitachi Medical Corporation, Tokyo, Japan).

Analyses of variance were performed to examine treatment (with two levels, i.e. MPH and ATX) × condition (with two levels, i.e. pre- and post-treatment) interactions. The threshold for statistical significance was set at p < 0.05. Bonferroni-adjusted p values are reported (i.e. corrected for multiple comparisons). We used PASW Statistics18.0J for Windows (SPSS, Tokyo, Japan) for statistical analyses.

## Results

### Demographic data

The demographic characteristics of the study participants are presented in Table 1. The participant groups did not differ in terms of mean age, sex, FIQ, ADHD-RS-IV-J scores including the ARF, ARI and ARH subscale scores, and SCWC-3 scores (p > 0.125 for all 7 variables). We found significant differences in the SCWC-1, SCWC-2 scores between the MPH and ATX groups (t = −2.52, p = 0.018; t = −2.53, p = 0.017).

### Correlation between Stroop task performance and participant characteristics

Because the MPH and ATX groups varied considerably in terms of SCWC-1 SCWC-2 scores, we calculated Spearman’s correlations for the SCWC scores, age, FIQ, and ADHD-RS-IV-J, as shown in Table 2. In the ATX group, the SCWC-1, SCWC-2 and SCWC-3 scores were positively correlated with age (ρ = 0.866, p < 0.000, ρ = 0.798, p < 0.001 and ρ = 0.718, p < 0.004), while none of SCWC scores significantly correlated with FIQ and ADHD-RS-IV-J scores. In the MPH group, the SCWC2 score were positive correlated with age (ρ = 0.522, p < 0.038), and SCWC1 score were positive correlated with FIQ (ρ = 0.557, p < 0.025), whereas none of the SCWC scores were significantly associated with ADHD-RS-IV-J scores.

**Table 2:** Correlation between Stroop task performance and participant characteristics

	MPH (n = 16)			ATX (n = 14)		
	SCWC-1	SCWC-2	SCWC-3	SCWC-1	SCWC-2	SCWC-3
Age	0.483	0.522*	0.417	0.866**	0.798**	0.718**
FIQ	0.557*	0.304	0.307	−0.159	−0.023	0.982
ARF	−0.368	−0.292	−0.351	−0.248	−0.237	−0.306
ARI	−0.001	−0.076	−0.162	−0.085	−0.116	−0.227
ARH	−0.448	−0.364	−0.335	−0.422	−0.376	−0.402

*MPH* methylphenidate, *ATX* atomoxetine, *FIQ* full-scale IQ, *WISC*-*IV* Wechsler Intelligence Scale for children-fourth edition, *ARF* ADHD RS IV-J full scores, *ARI* ADHD RS IV-J inattention subscale scores, *ARH* ADHD RS IV-J hyperactivity subscale scores, *SCWC*-*1* Stroop color-word task number of correct answers first time, *SCWC*-*2* Stroop color-word task number of correct answers second time, *SCWC*-*3* Stroop color-word task number of correct answers third time

* p < 0.05

** p < 0.01

### Clinical and behavioral improvement

Both treatments were associated with statistically significant improvements in terms of both ADHD-RS-IV-J scores and SCWC scores, as shown in Table 3. In both groups, the ADHD-RS-IV-J scores including the ARF, ARI and ARH subscale scores in the post-treatment condition were significantly lower than scores in the pre-treatment condition (p < 0.01 for all 6 variables). Additionally, the SCWC-1, SCWC-2 and SCWC-3 scores in the post-treatment condition were significantly higher than those in the pre-treatment condition (p < 0.033 for all 6 variables). There were no significant main effects of treatment condition × medication interactions for any of the performance measures (p > 0.098 for all 6 variables).

**Table 3:** Clinical outcome and task performance

	MPH (n = 16)		ATX (n = 14)		p value		
	Pre-	Post-	Pre-	Post-	MPH	ATX	
	Mean (SD)	Mean (SD)	Mean (SD)	Mean (SD)	Pre vs post^a^	Pre vs post^a^	Time × Drug Interaction^b^
ARF	30.63 (10.65)	17.06 (10.51)	32.29 (13.46)	22.71 (10.54)	0.000	0.001	0.208
ARI	16.75 (5.52)	10.13 (6.01)	18.29 (6.84)	13.71 (6.27)	0.000	0.010	0.272
ARH	13.88 (6.72)	6.94 (5.47)	14.00 (7.99)	9.00 (4.84)	0.000	0.002	0.295
SCWC-1	18.31 (7.66)	27.75 (11.70)	25.86 (8.76)	33.79 (13.57)	0.000	0.000	0.520
SCWC-2	19.81 (9.56)	28.13 (10.54)	29.36 (11.11)	33.50 (12.82)	0.000	0.033	0.098
SCWC-3	21.19 (9.17)	26.44 (10.91)	25.36 (10.35)	34.07 (12.16)	0.006	0.002	0.210

*MPH* methylphenidate, *ATX* atomoxetine, *ARF* ADHD RS IV-J full scores, *ARI* ADHD RS IV-J inattention subscale scores, *ARH* ADHD RS IV-J hyperactivity subscale scores, *SCWC*-*1* Stroop color-word task number of correct answers first time, *SCWC*-*2* Stroop color-word task number of correct answers second time, *SCWC*-*3* Stroop color-word task number of correct answers third time

^a^Two-tailed paired t test

^b^Two-way factorial ANOVA

### Comparison of NIRS measurements between pre- and post- treatment

**Fig. 2:**
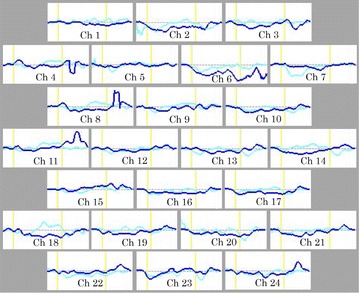
Grand average waveforms showing changes in oxyhemoglobin(oxy-Hb) during the Stroop color-word task pre- and post-MPH. *Cyan lines* indicate pre-MPH and *blue lines* indicate post-MPH. *Yellow lines* indicate the beginning and end of each trial. *Ch* channel

**Fig. 3:**
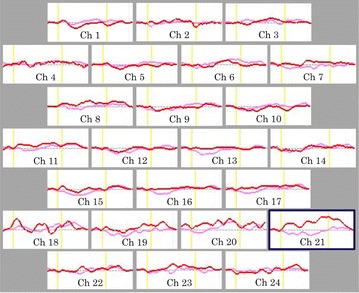
Grand average waveforms showing changes in oxyhemoglobin(oxy-Hb) during the Stroop color-word task pre- and post-ATX. *Pink lines* indicate pre-ATX and *red lines* indicate post-ATX. *Yellow lines* indicate the beginning and end of each trial. The statistically significant region is shown within *navy frames* (Ch21). *Ch* channel

We calculated the grand average waveforms of [oxy-Hb] concentration changes during the Stroop color-word task in the pre- and post-treatment condition (Figs. 2, 3).

In the MPH group, the grand waveforms of [oxy-Hb] concentration showed little change in both pre- and post-conditions (Fig. 2). We did not find any differences in mean [oxy-Hb] measurements between pre- and post-MPH in any of the 24 channels that were recorded in Table 4. By contrast, in the ATX group, the grand waveforms of [oxy-Hb] concentration change appeared to increase substantially during task performance in the post- rather than in the pre-condition (Fig. 3). On channel 21, the mean oxy-Hb measurement was significantly larger in the post-condition relative to the pre-condition, as displayed in Table 5.

**Table 4:** Difference in mean oxyhemoglobin between the task and post-task periods pre- and post-MPH

	Pre-MPH (mMmm)		Post-MPH (mMmm)		Student’s t test
	Mean	SD	Mean	SD	
Ch1	0.0136	0.1101	−0.0319	0.1463	NS
Ch2	−0.0254	0.1011	−0.0739	0.0782	NS
Ch3	−0.0047	0.0878	−0.0689	0.1241	NS
Ch4	0.0097	0.0759	−0.0062	0.0981	NS
Ch5	−0.0209	0.0746	−0.0225	0.0585	NS
Ch6	−0.0245	0.1071	−0.1513	0.2498	NS
Ch7	−0.0354	0.1242	−0.0337	0.1116	NS
Ch8	0.0019	0.0623	−0.0018	0.1245	NS
Ch9	−0.0009	0.0707	−0.0173	0.1256	NS
Ch10	−0.0601	0.1518	−0.0436	0.0936	NS
Ch11	0.0174	0.0686	0.0426	0.2025	NS
Ch12	−0.0092	0.0494	−0.013	0.1055	NS
Ch13	−0.0371	0.0739	−0.023	0.0948	NS
Ch14	−0.0001	0.1577	−0.0349	0.1056	NS
Ch15	−0.0033	0.0667	0.0233	0.2365	NS
Ch16	−0.0356	0.0745	−0.0476	0.0712	NS
Ch17	−0.0154	0.0741	−0.0415	0.0804	NS
Ch18	0.0207	0.1593	−0.0691	0.1423	NS
Ch19	0.0006	0.138	−0.035	0.1215	NS
Ch20	−0.0571	0.091	−0.0582	0.1153	NS
Ch21	−0.00569	0.1034	−0.0432	0.1346	NS
Ch22	−0.0553	0.0996	−0.0371	0.0891	NS
Ch23	−0.0369	0.0936	−0.037	0.0688	NS
Ch24	−0.0414	0.0836	−0.0326	0.1047	NS

Group differences tested with t test

*NS* not significant

**Table 5:** Difference in mean oxyhemoglobin measurements between the task and post-task periods pre- and post-ATX

	Pre-ATX (mMmm)		Post-ATX (mMmm)		Student’s t test	Bonferroni correction
	Mean	SD	Mean	SD		
Ch1	0.013	0.0717	−0.0389	0.0706	NS	NS
Ch2	−0.0092	0.1313	−0.0309	0.0681	NS	NS
Ch3	0.0294	0.0837	0.0194	0.0848	NS	NS
Ch4	0.0259	0.0595	0.018	0.0571	NS	NS
Ch5	−0.0008	0.0656	−0.0191	0.0542	NS	NS
Ch6	0.0213	0.0789	0.0000	0.0477	NS	NS
Ch7	−0.0209	0.0634	0.0133	0.0821	NS	NS
Ch8	0.0107	0.0767	0.0508	0.0761	NS	NS
Ch9	−0.0049	0.0734	−0.0095	0.0968	NS	NS
Ch10	−0.0182	0.0419	−0.0312	0.0667	NS	NS
Ch11	0.0042	0.0941	0.0483	0.0747	NS	NS
Ch12	−0.0337	0.0558	−0.0159	0.0621	NS	NS
Ch13	−0.0363	0.0865	−0.0059	0.0565	NS	NS
Ch14	−0.0039	0.0695	0.0042	0.0731	NS	NS
Ch15	−0.0278	0.0949	0.0061	0.084	NS	NS
Ch16	−0.0119	0.1077	0.0109	0.0606	NS	NS
Ch17	−0.0013	0.057	0.0314	0.0438	NS	NS
Ch18	0.046	0.0746	0.0456	0.1136	NS	NS
Ch19	−0.0391	0.0727	0.0178	0.0652	p = 0.049	NS
Ch20	0.0585	0.0871	0.0883	0.1638	NS	NS
Ch21	−0.0341	0.0753	0.0956	0.0954	p = 0.000	*
Ch22	0.006	0.0844	0.0156	0.0813	NS	NS
Ch23	−0.0101	0.0803	0.0202	0.0899	NS	NS
Ch24	0.006	0.101	−0.0123	0.0773	NS	NS

Group differences tested with t test

*NS* not significant

* Significant with Bonferroni correction for multiple comparisons

### Comparison of NIRS measurements between two groups

Channel 21 showed significant treatment-by-condition interactions (F = 13.102, p = 0.002). However, there were no main effects for either treatment or condition on channel 21 (F = 2.260, p = 0.147; F = 3.99, p = 0.058). We did not find any differences in mean [oxy-Hb] measurements between the pre-ATX and the pre-MPH (t = 0.756, p = 0.458) on this channel. However, the mean oxy-Hb measurement for channel 21 was significantly larger for post-ATX relative to post-MPH (t = −0.2802, p = 0.009).

### Correlation between degree of clinical improvement and hemodynamic change in Channel 21

We conducted Spearman’s rank correlation analyses between hemodynamic change in channel 21 with scores of SCWC and ADHD-RS-IV-J scores, shown in Table 6. There were no correlations between hemodynamic change and these scores for either ATX or MPH (all p > 0.2).

**Table 6:** Correlation between degree of clinical improvement and hemodynamic change in channel 21

	MPH	ATX
ARF	−0.006	−0.196
ARI	−0.072	−0.406
ARH	−0.017	−0.087
SCWC-1	−0.331	0.147
SCWC-2	−0.213	−0.014
SCWC-3	−0.349	−0.100

Tested using Spearman’s correlation test

*MPH* methylphenidate, *ATX* atomoxetine, *ARF* ADHD RS IV-J full scores, *ARI* ADHD RS IV-J inattention subscale scores, *ARH* ADHD RS IV-J hyperactivity subscale scores, *SCWC*-*1* Stroop color-word task number of correct answers first time, *SCWC*-*2* Stroop color-word task number of correct answers second time, *SCWC*-*3* Stroop color-word task number of correct answers third time

All p > 0.216

## Discussion

To our knowledge, this is the first NIRS study to compare the effectiveness of MPH with ATX directly in children with ADHD by measuring hemodynamic responses during the Stroop color-word task. ATX significantly increased activation in the prefrontal cortex, especially in left lateral frontal pole cortex (FPC), after 12 weeks of administration. MPH did not increase activation in the prefrontal cortex, but it did make comparable improvements in terms of ADHD symptoms and Stroop color-word task performance to those seen in ATX.

Some studies have referred indirectly to differences in the neurobiological actions between MPH and ATX. Event-related potential studies of oddball tasks in pediatric ADHD have shown that MPH can normalize low P300 or mismatch negativity amplitudes [41], while ATX can normalize long P300 latencies and low MMN amplitudes, at least partially [42]. In a fMRI study of adult ADHD using a multi-source interference task, ATX did not activate dorsal anterior midcingulate cortex [43], as MPH has been shown to do [44]. However, few studies have directly investigated how the pharmacological mechanisms of action differ between the two drugs, and little is known about the mechanisms by which they exert their therapeutic effects. In one fMRI study that used a go/no-go task with 36 participants with pediatric ADHD, comparable improvements in response inhibition and ADHD symptoms were seen after 6 to 8 weeks of daily treatment with MPH vs ATX. Symptomatic improvement was associated with gains in task-related activation for ATX and reductions in activation for MPH in the right inferior frontal gyrus, left anterior cingulate/supplementary motor area, and bilateral posterior cingulate cortex [45]. In another fMRI study using a counting Stroop paradigm, 12 weeks of ATX pharmacotherapy decreased activity in the dorsal anterior cingulate cortex and dorsolateral prefrontal cortex in 42 participants with pediatric ADHD, which correlated with improvement in focused attention. In contrast, MPH increased activity in the inferior frontal gyrus, which correlated with decreasing severity of impulsivity [46]. Comparing effects of acute doses of both drugs and a placebo with boys with ADHD during a stop task, MPH had a drug-specific effect of normalizing the right ventrolateral prefrontal and cerebellar under-activation observed under both placebo and ATX [47]. Taken together, these reports indicate that the mechanisms by which MPH and ATX exert their therapeutic effects are different: this is consistent with the findings from the present study. Nevertheless, the concept of drug-specific laterality effects on prefrontal regions is still controversial. Our data showed that ATX upregulated the frontal cortex during Stroop interference, at least partially. The present findings suggest that frontal mechanisms serve an important role in the therapeutic actions of ATX. However, despite the fact that MPH did not increase activation in the PFC, there were still comparable improvements in terms of ADHD symptoms for those taking this medication. One parsimonious explanation is that MPH increases activation in other brain regions, which might contribute to the improvement in ADHD symptoms.

Volkow et al. [48, 49] found that in healthy adults, MPH enhanced the salience of a reward task, increased levels of extra-cellular dopamine, and induced reductions in glucose metabolism within the default mode network (DMN). The DMN is a distributed brain system, comprising medial pre-frontal cortex and medial and lateral parietal regions. It is anti correlated with the attentional networks activated by goal-directed behavior, and is thought to reflect intrinsic activity [50]. Recently, influential new brain network models [51, 52] have proposed that proper sustained attention functioning requires both engagement of task-positive networks (TPNs), including a frontoparietal control network and dorsal and ventral attention networks, and suppression of the DMN [50, 53, 54]. A failure of the anti-phase synchronization between DMN and TPN may be involved in the manifestation of ADHD. There is evidence suggesting that the striatal DA system plays a role in the modulation of the DMN [55, 56]. MPH produces robust increases in extracellular dopamine levels [57], which potentiate corticostriatal inputs [58] and have been found to enhance striatal activation in child ADHD [59, 60]. Furthermore, some studies have shown that MPH may normalize DMN deactivation patterns [61, 62]. Therefore, we speculate that MPH might tend to activate DMN regions rather than TPN during task-related activation. In contrast, an increase of prefrontal activation has been reported after MPH treatment in several studies using different neuroimaging modalities, including NIRS [44, 59, 63, 64]. The variability in findings across studies is likely related to different cognitive tasks, dosage, patients’ ages, and treatment duration.

Increases in left lateral FPC activity were observed after ATX treatment in our study. However, we found no significant correlations between the hemodynamic changes in this area and degree of the clinical improvements. The FPC is the most anterior part of the cerebral cortex, and has reciprocal connections with most prefrontal areas [65, 66]. Tsujimoto et al. suggested that the FPC has a role in monitoring and evaluating decisions, especially those with a self-generational component [67]. Arai et al. found that children with ADHD show abnormalities in functional maturation of the frontal pole [68]. Based on these findings, a direction for future research will be to assess participants using another battery associated with self-generated behavior, separate to NIRS recordings.

The results of the present study suggest that multi-channel NIRS systems may have potential in the pharmacotherapeutic evaluation in children with ADHD for clinical practice. It is very significant for patients that an effect of the pharmacotherapy is visualized. In the future, it is need to predict the effect of the pharmacotherapy using the NIRS for clinical practice.

The present study has several potential limitations. First, methodological limitations include the relatively small number of participants, non-randomised study, and lack of a double-blind, placebo-controlled design. At baseline, the ATX group had higher mean SCWC1 and SCWC2 scores than the MPH group. Although scores were not correlated with degree of clinical severity with ADHD-RS, the two groups were not quite entirely equivalent in their characteristics. Future work seeking to compare MPH, ATX and/or placebo should consider a double-blind randomized or crossover design with larger samples. Second, we had no healthy control as a comparison cohort. Our study showed that ATX significantly increased activation in channel 21. In one previous NIRS study using the Stroop, Negoro et al. reported a lower increase of oxy-changes in channels 8, 18, 19, 21, and 22 in individuals with child ADHD compared with controls [23]. Considering the above findings, we predicted that improvement of ADHD symptoms with ATX treatment would be associated with increased activation in those regions; our findings were consistent with these predictions. Third, NIRS does not detect activity in deeper cortical structures, such as the medial pre-frontal cortex, which is part of the DMN. Fourth, the spatial resolution for the detection of hemodynamic responses from the scalp surface using NIRS is lower than that of fMRI, SPECT, or PET. However, the spatial resolution may be within an acceptable range because previous NIRS studies have also found clear distinctions in hemodynamic responses between diagnostic groups [23–26, 28, 69].

## Conclusions

In conclusion, this is the first NIRS study using the Stroop interference task to examine how the pharmacological mechanisms of action differ between MPH and ATX. Findings suggest that effective treatment with MPH and ATX is produced by distinct mechanisms in frontal regions.

## Abbreviations

MPH: methylphenidate
ATX: atomoxetine
ADHD: attention deficit/hyperactivity disorder
fMRI: functional magnetic resonance imaging
NIRS: near-infrared spectroscopy
DA: dopamine
NE: norepinephrine
PFC: prefrontal cortex
SPECT: single-photon emission computed tomography
PET: positron emission tomography
FIQ: full-scale IQ
WISC-IV: Wechsler intelligence scale for children-fourth edition
ARF: ADHD RS IV-J full scores
ARI: ADHD RS IV-J inattention subscale’s scores
ARH: ADHD RS IV-J hyperactivity subscale’s scores
SCWC-1: Stroop color-word task number of correct answers first time
SCWC-2: Stroop color-word task number of correct answers second time
SCWC-3: Stroop color-word task number of correct answers third time
FPC: frontal pole cortex
DMN: default mode network
TPNs: task-positive networks
